# Status of prehospital delay and intravenous thrombolysis in the management of acute ischemic stroke in Nepal

**DOI:** 10.1186/s12883-019-1378-3

**Published:** 2019-07-09

**Authors:** Gaurav Nepal, Jayant Kumar Yadav, Babin Basnet, Tirtha Man Shrestha, Ghanshyam Kharel, Rajeev Ojha

**Affiliations:** 1 Medical Student, Tribhuvan University Institute of Medicine, Maharajgunj, Kathmandu, Nepal; 2 Department of General Practice and Emergency Medicine, Tribhuvan University Institute of Medicine, Maharajgunj, Kathmandu, Nepal; 3 Department of Neurology, Tribhuvan University Institute of Medicine, Maharajgunj, Kathmandu, Nepal

**Keywords:** Stroke, Ischemic stroke, Pre-hospital delay, rtPA, Thrombolysis, Nepal

## Abstract

**Background:**

Intravenous thrombolysis has been recently introduced in Nepal for the management of acute ischemic stroke. Pre-hospital delay is one of the main reasons that hinder thrombolytic therapy. The objective of this study was to evaluate the status of prehospital delay and thrombolysis in Nepal.

**Methods:**

Data were prospectively collected from patients of both genders, age >  18 years who arrived at the emergency department (ED) with symptoms and neuroimaging findings consistent with an ischemic stroke. Patient data were obtained from ED form and standard questionnaires were used to assess factors resulting in prehospital delay. Modified Rankin scale and National Institute of Health stroke scale were used to assess the degree of disability and severity of stroke respectively.

**Results:**

A total of 228 patients were enrolled in the study between August 2017 and August 2018. Only 46 (20.17%) patients arrived within the time frame for thrombolysis. Onset at daytime (OR: 4.07; 95% CI: 1.65–10.1; *p* = 0.001), stroke symptoms facial deviation (OR: 5.03; 95% CI: 2.47 to 10.26; *p* = 0.000) and speech disturbances (OR: 2.34; 95% CI: 1.06 to 5.1; *p* = 0.021), identification of stroke (OR: 22.36; 95% CI: 9.42–53.04;*p* = 0.000), rushing to ED after onset of symptoms (OR: 2.93; 95% CI: 1.5–5.7; *p* = 0.001), awareness of treatment of stroke (OR: 10.21; 95% CI: 4.8–21.6; *p* = 0.000), direct presentation (OR: 4.2; 95% CI: 2.09–8.66; *p* = 0.000), the distance less than 20 km (OR: 7.9; 95% CI: 3.8–16.5; *p* = 0.000), and education above high school (OR:4.85; 95% CI: 2.2–10.5; *p* = 0.000) were associated with early arrival. Heavy traffic, income below 1000 USD per annum and diabetes mellitus were associated with delayed arrival to ED. Out of 46 early arrival patients, only 30 patients (13.15%) received tissue plasminogen activator during the study period, while others were deprived because of their inability to afford the treatment cost.

**Conclusion:**

Community-based intervention to spread awareness, establishing comprehensive stroke centers, training specialists, improving emergency services, establishment of telestroke facilities and encouraging the use of low-cost tenecteplase as an alternative to alteplase can help improve care for stroke patients in Nepal.

## Background

According to the Global Burden of Disease Study, stroke is the second leading cause of death globally and the third leading cause of premature death and disability as measured in Disability Adjusted Life Years (DALY) [1]. Stroke also accounts for 4.1% of total global DALY producing immense health and economic burden globally [1, 2]. In 2013, stroke accounted for 6.5millions deaths worldwide of which 51% had suffered ischemic stroke. Similarly, 113 million DALYs were due to the stroke of which ischemic stroke comprised 58%. In the same year, total new stroke cases recorded was 10.3 million of which 67% were ischemic type [2]. The majority of the burden of stroke continues to reside in developing countries comprising 75.2% of deaths from stroke and 81.0% of stroke-related DALYs [2]. According to data of the central referral hospital of Nepal, ischemic stroke accounted for 36.5% of all admitted cases in neurology ward and 73.8% of all admitted cases of stroke [3].

Until now, intravenous thrombolysis (IVT) with tissue plasminogen activator (t-PA) within 4.5 h and endovascular thrombectomy within 6 h are the only approved treatments for acute ischemic stroke [4, 5]. The use of IVT for the management of ischemic stroke was introduced in Nepal in 2012 [6]. Endovascular thrombectomy is one of the recent advances in the treatment of ischemic stroke but has its own limitations. It is reserved for ischemic stroke of proximal large arteries in the anterior circulation but requires extensive setup and expertise which is not currently feasible in a limited resource setting of Nepal. Thus, IVT appears to be a better option for ischemic stroke patients in Nepal.

IVT has a very narrow therapeutic window and early arrival to the hospital for IVT with t-PA is of paramount importance for acute ischemic stroke management. However, only a small proportion of patients with ischemic stroke receive reperfusion therapy with IVT for which delayed presentation to hospitals is one of the most important limiting factors. Further, early thrombolysis is associated with good clinical outcomes compared to late thrombolysis even within therapeutic time window. Hence the benefit is strongly time-dependent [7, 8]. To the best of our knowledge, there has been no study of prehospital delays in patients with acute ischemic stroke in Nepal. We, therefore, sought to evaluate the factors associated with delayed presentation to the hospital among patients who suffered an acute stroke in Nepal and the status of thrombolysis in Nepal.

## Methods

This study was approved by the Institutional Review Board of Tribhuvan University, Institute of Medicine. The approval number for our study is 31(6–11-E) ^2^/074/075. Informed written consent was obtained from the patients themselves, where possible, and their accompanying family members. The site of patient enrollment was Tribhuvan University Teaching Hospital (TUTH) located in capital city Kathmandu. With 700-bed and 32 departments, it is the largest hospital in the country and tertiary referral center for all kinds of disease and conditions including stroke.

### Inclusion criteria

Between August 2017 and August 2018, data were prospectively collected from patients of both sexes, age >  18 years old, who arrived at the emergency department (ED) of TUTH with signs and symptoms corresponding to ischemic stroke and confirmed by neuroimaging.

### Exclusion criteria

We excluded the patients who suffered from an ischemic stroke when in our hospital, patients with contraindication to thrombolysis, those having a hemorrhagic stroke and presenting 7 days after stroke symptoms, and patients with unknown duration of symptom onset. We excluded all the patients coming to our ED from places outside Kathmandu to prevent selection bias.

### Variables

Time of stroke onset was defined as the time the patient or an observer first noted a neurological deficit. If the symptoms were present on waking, it was assumed that stroke had occurred during the night and the time of onset was taken when the patient was last seen without symptoms. The exact time of arrival at hospital was extracted from emergency room form. A standard structured questionnaire was filled by interviewing the patient (if possible) and family members/relatives after taking informed written consent. The questionnaire documented the patient’s age, sex, educational level, the day of onset, financial status, symptoms, ability to identify stroke, past history, distance from the site where the event happened, response to symptoms, referral, patient’s awareness of stroke treatment, and the mode of transportation. The modified Rankin Scale (mRS) was used for assessing the degree of disability [9]. The National Institute of Health Stroke Scale (NIHSS) was used for assessing stroke severity: mild (1–5), moderately severe (6–14), severe (15–24) and very severe (> 25) [10].

A threshold of 3 h was used to classify patients into two groups, i.e., arrival to the ED within 3 h after the onset of symptoms (non-delayed/early) or arrival beyond this time (delayed/late) [11]. The day of onset was divided into weekdays and holidays (Saturday, public holidays) and the time of onset was divided into daytime (from 7 am to 7 pm) and night-time (7 pm to 7 am) as they impact the arrival time. The site where the acute event happened was divided in the home and outside home and witness of symptoms onset was categorized into patient and others. Transportation variables included travel to the hospital by ambulance or by other methods (motorcycle, car, taxi).

Symptoms at onset included: Facial deviation or weakness, visual disturbance, limb weakness, speech disturbance, sensory disturbance, headache, and unconsciousness. Hypertension, diabetes, cardiovascular diseases, smoking, alcohol consumption and history of stroke were measured as risk factors for ischemic stroke.

### Statistical analysis

Arrival time to ED after stroke was the dependent variable dichotomized as either early (≤3 h) or late (> 3 h). Various other variables, as mentioned above, were independent variables. Univariate analyses (independent sample t-test for continuous variables, and χ2 test for categorical variables, as appropriate) were performed to assess the association of each of the independent variables and dependent variable. Explanatory variables, which were identified by univariate analysis at *P* < 0.2, were selected and entered into a binary logistic regression model to identify predictors of an early arrival. All the statistical analyses were performed using SPSS 21 (IBM Corp. Released 2012. IBM SPSS Statistics for Windows, Version 21.0. Armonk, NY: IBM Corp.). For all statistical analysis, significance was accepted at *P* < 0.05.

## Results

### Patient selection

**Fig. 1:**
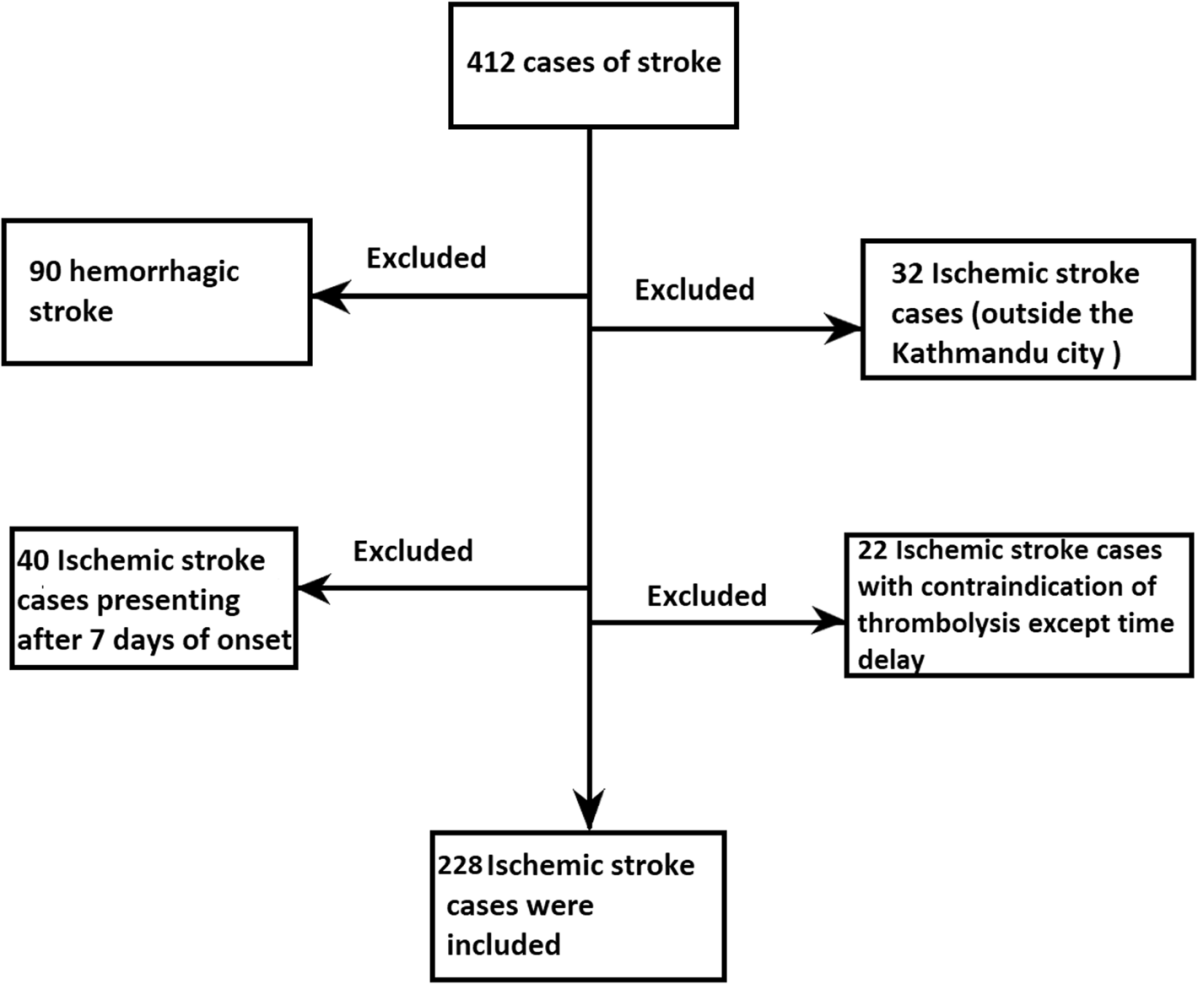
Flow diagram of patient selection

During the study period, 412 patients arrived in the emergency department (ED) with stroke. Of the 412 stroke patients, 90 patients were excluded because plain CT head showed intracerebral hemorrhages. Among 322 ischemic stroke patients, 32 patients were excluded because they were coming from places outside Kathmandu city and 40 patients were excluded as they presented 7 days after the onset of symptoms. Likewise, twenty-two ischemic stroke patients were found to have contraindication to thrombolysis and were thus excluded. Finally, 228 ischemic stroke cases were included in our study as shown in Fig. 1.

### Patient characteristics

There were a total of 228 ischemic stroke patients included in our study, 121(53.1%) of them were male and 154 (67.5%) were above the age of 60. On the basis of NIHSS, 62 (27.2%) patients had a mild stroke, 140(61.2%) had a moderately severe stroke, 19(8.3%) had a severe stroke, and 7(3.1%) had a very severe stroke. On the basis of mRS, 28 (12.3%) patients had no significant disability, 53(23.2%) had a slight disability, 30(13.2%) had a moderate disability, 59(25.9%) had a moderately severe disability, and 58(25.4%) had a severe disability. In terms of symptoms at onset, 94 patients (41.2%) developed facial deviation or weakness, 23 patients (10.1%) developed visual disturbances, 154 patients (67.5%) developed limb weakness, 153 patients (67.1%) developed speech disturbances, 11 patients (4.8%) developed sensory disturbances, 39 patients (17.1%) had headache, and 34 patients (14.9%) became unconscious. According to patient’s history, hypertension was found in 128 patients (56.1%), diabetes in 40 patients (17.5%), smoking in 66 patients (28.9%), significant alcohol consumption in 62 patients (27.2%), coronary heart disease in 21 patients (9.2%), and previous stroke in 27(11.8%). All other patient characteristics are tabulated in Table 1.

**Table 1:** Baseline characteristics of the study population

Characteristics		N (%)
Age	< 60	74 (32.5)
	> 60	154 (67.5)
Sex	Male	121 (53.1)
	Female	107 (46.9)
Arrival	Early	46 (20.17)
	Late	182 (79.83)
Time of onset	7 am-7 pm	153 (67.1)
	7 pm-7 am	75 (32.9)
Day of onset	Weekday	183 (80.3)
	Holiday	45 (19.7)
Location	Home	192 (84.2)
	Outside home	36 (15.8)
Identification of stroke	Yes	36 (15.8)
	No	192 (84.2)
Facial palsy	Yes	94 (41.2)
	No	134 (58.5)
Visual disturbances	Yes	23 (10.1)
	No	205 (89.9)
Limb weakness	Yes	154 (67.5)
	No	74 (32.5)
Speech disturbances	Yes	153 (67.1)
	No	75 (32.9)
Sensory	Yes	11 (4.8)
	No	217 (95.2)
Headache	Yes	39 (17.1)
	No	189 (82.9)
Unconsciousness	Yes	34 (14.9)
	No	194 (57.9)
Response of symptoms	Rush to emergency	96 (42.1)
	Wait for symptoms to subside	132 (57.9)
Awareness of stroke treatment	Yes	44 (19.3)
	No	184 (80.7)
Presentation to ER	Direct	101 (44.3)
	Referral	127 (55.7)
Transportation	Ambulance	70 (30.7)
	Others	158 (69.3)
Traffic jam	Present	107 (46.9)
	Not present	121 (53.1)
Distance from hospital	< 20 KM	82 (36)
	> 20 KM	146 (64)
Education	Above high school	34 (14.9)
	Below high school	194 (85.1)
Income	> 1 lacks per annum	106 (46.5)
	< 1 lacks per annum	122 (53.5)
Hypertension	Yes	128 (56.1)
	No	100 (43.9)
Diabetes mellitus	Yes	40 (17.5)
	No	188 (82.5)
Smoking	Yes	66 (28.9)
	No	162 (71.1)
Alcohol consumption	Yes	62 (27.2)
	No	166 (72.8)
Coronary arterial disease	Yes	21 (9.2)
	No	207 (90.8)
History of stroke	Present	27 (11.8)
	Not present	201 (88.2)
Treatment	R-tPA	30 (13.15)
	Aspirin	198 (86.84)

### Univariate analysis of factors associated with prehospital delay

The early group had 46 patients (20.2%) and the late group had 182 patients (79.8%). In the univariate analysis (Table 2), onset at daytime (OR: 4.07; 95% CI: 1.65 to 10.1; *p* = 0.001), stroke symptoms facial deviation (OR: 5.03; 95% CI: 2.47 to 10.26; *p* = 0.000) and speech disturbances (OR: 2.34; 95% CI: 1.06 to 5.1; *p* = 0.021), identification of stroke (OR: 22.36; 95% CI: 9.42 to 53.04; *p* = 0.000), rushing to ED after onset of symptoms (OR: 2.93; 95% CI: 1.5 to 5.7; *p* = 0.001), awareness of treatment of stroke (OR: 10.21; 95% CI: 4.8 to 21.6; *p* = 0.000), direct presentation (OR: 4.2; 95% CI: 2.09 to 8.66; *p* = 0.000), the distance less than 20 km (OR: 7.9; 95% CI: 3.8 to 16.5; *p* = 0.000), and education above high school (OR:4.85; 95% CI: 2.2 to 10.5; *p* = 0.000) were associated with early arrival. Presence of heavy traffic, income below 1000 USD per annum and diabetes mellitus were associated with delayed arrival to ED. Age, sex, date, location, limb weakness, visual impairment, sensory disturbances, unconsciousness, headache, transportation, hypertension, smoking, alcohol consumption, coronary heart disease, and past history of stroke did not reach statistical significance. The mean NIHSS and mRS did not differ between the two groups.

**Table 2:** Association between study variables and early arrival at emergency department

Study variables		Early n (%)	Late n (%)	Odds ratio (OR)	95% C.I.	*P* value
Age	< 60	18 (39.1)	56 (30.8)	1.44	0.74–2.8	0.18
	> 60	28 (60.9)	126 (69.2)	1	Reference	
Sex	Male	25 (54.3)	96 (52.7)	1.066	0.56–2.04	0.49
	Female	21 (45.7)	86 (47.3)	1	Reference	
Time of onset	7 am-7 pm	40 (87)	113 (62.1)	4.07	1.65–10.1	0.001
	7 pm-7 am	6 (13)	69 (37.9)	1	Reference	
Day of onset	Weekday	41 (89.1)	142 (78)	2.31	0.85–6.23	0.064
	Holiday	5 (10.9)	40 (22)	1	Reference	
Location	Home	42 (91.3)	150 (82.4)	2.24	0.75–6.6	0.101
	Outside home	4 (8.7)	32 (17.6)	1	Reference	
Identification of stroke	Yes	26 (56.5)	10 (5.5)	22.36	9.42–53.04	0.000
	No	20 (43.5)	172 (94.5)	1	Reference	
Facial palsy	Yes	33 (71.7)	61 (33.5)	5.03	2.47–10.26	0.000
	No	13 (28.3)	121 (66.5)	1	Reference	
Visual disturbances	Yes	3 (6.55)	20 (11)	0.563	0.16–1.99	0.276
	No	43 (93.45)	162 (89)	1	Reference	
Limb weakness	Yes	32 (69.6)	122 (67)	1.124	0.56–2.26	0.445
	No	14 (30.4)	60 (33)	1	Reference	
Speech disturbances	Yes	37 (80.4)	116 (63.7)	2.34	1.06–5.1	0.021
	No	9 (19.6)	66 (36.3)	1	Reference	
Sensory	Yes	1 (2.2)	10 (5.5)	0.382	0.05–3.06	0.31
	No	45 (97.8)	172 (94.5)	1	Reference	
Headache	Yes	10 (21.7)	29 (15.9)	1.46	0.65–3.27	0.233
	No	36 (78.3)	153 (84.1)	1	Reference	
Unconsciousness	Yes	3 (6.5)	31 (17)	0.34	0.1–1.1	0.053
	No	43 (93.5)	151 (83)	1	Reference	
Response of symptoms	Rush to emergency	29 (63)	67 (36.8)	2.93	1.5–5.7	0.001
	Wait for symptoms to subside	17 (37)	115 (63.2)	1	Reference	
Awareness of stroke treatment	Yes	25 (54.3)	19 (10.4)	10.21	4.8–21.6	0.000
	No	21 (45.7)	163 (89.6)	1	Reference	
Presentation to ER	Direct	33 (71.7)	68 (37.4)	4.2	2.09–8.66	0.000
	Referral	13 (28.3)	114 (62.6)	1	Reference	
Transportation	Ambulance	19 (41.3)	51 (28)	1.8	0.92–3.5	0.06
	Others	27 (28.7)	131 (72)	1	Reference	
Traffic jam	Present	10 (21.7)	97 (53.3)	0.243	0.11–0.52	0.000
	Not present	36 (78.3)	85 (46.7)	1	Reference	
Distance from hospital	< 20 KM	34 (73.9)	48 (26.4)	7.9	3.8–16.5	0.000
	> 20 KM	12 (26.1)	134 (73.6)	1	Reference	
Education	Above high school	16 (34.8)	18 (9.9)	4.85	2.2–10.5	0.000
	Below high school	30 (65.2)	164 (90.1)	1	Reference	
Income	< 1 lacks per annum	32 (69.6)	90 (49.5)	0.42	0.21–0.85	0.011
	> 1 lacks per annum	14 (30.4)	92 (50.5)	1	Reference	
Hypertension	Yes	29 (63)	99 (54.4)	1.43	0.73–2.7	0.187
	No	17 (37)	83 (45.6)	1	Reference	
Diabetes mellitus	Yes	3 (6.5)	37 (20.3)	0.273	0.08–0.9	0.018
	No	43 (93.5)	145 (79.7)	1	Reference	
Smoking	Yes	11 (23.9)	55 (30.2)	0.72	0.35–1.5	0.257
	No	35 (76.1)	127 (69.8)	1	Reference	
Alcohol consumption	Yes	15 (32.6)	47 (25.8)	1.4	0.7–2.8	0.228
	No	31 (63.4)	135 (74.2)	1	Reference	
Coronary arterial disease	Yes	5 (10.9)	16 (8.8)	1.26	0.43–3.6	0.422
	No	41 (89.1)	166 (91.2)	1	Reference	
History of stroke	Present	9 (19.6)	18 (9.9)	2.21	0.92–5.3	0.06
	Not present	37 (80.4)	164 (90.1)	1	Reference	
Baseline score at admission (mean ± SD)	mRS	3.02 ± 1.42	3.36 ± 1.37	NA	NA	0.143
	NIHSS	7.58 ± 3.87	8.06 ± 5.26	NA	NA	0.568

### Logistic regression analysis

To identify the independent predictors of a reduced prehospital delay (early arrival), we used the logistic regression model. Logistic regression showed that location inside home, rushing to the emergency after symptoms onset, absence of traffic jam, and income above 1000 USD were all independent predictors of a reduced prehospital delay, whereas failure to identify stroke, no awareness of stroke treatment, and distance > 20 km were associated with delayed arrival to ED (Table 3).

**Table 3:** Final logistic regression model results

Predictor variables	β estimate	Standard error	*P* value	Exp(B)	95% C.I. for EXP(B)
Location: Inside home	2.179	0.464	0.000	8.836	(3.559, 21.937)
Identification of stroke: No	−2.148	0.640	0.001	0.117	(0.033, 0.409)
Response of symptoms: Rush to emergency	1.336	0.591	0.024	3.805	(1.196, 12.107)
Awareness of stroke treatment: No	−1.961	0.614	0.001	0.141	(0.042, 0.469)
Traffic Jam: No	1.230	0.437	0.005	3.421	(1.453, 8.054)
Distance: > 20 KM	− 1.266	0.432	0.003	0.282	(0.121, 0.658)
Income above 1000 USD	1.259	0.427	0.003	3.521	(1.524, 8.136)

### Thrombolysis

Overall, only 46 patients arrived at the hospital early, out of which 30 patients (13.15%) received tissue plasminogen activator during the study period. Sixteen patients even after an early arrival were deprived of thrombolysis. None of the patients in the late arrival group received thrombolysis.

## Discussion

The results of our study suggest that there is considerable pre-hospital delay among patients with acute stroke in Nepal. Identification of stroke, awareness of stroke treatment, education above high school and rushing to the emergency after the onset of symptom all shortened the prehospital delay. Among all major symptoms of stroke, only facial deviation or weakness, and speech disturbances were associated with a shortened prehospital delay in our study. Symptoms like facial deformity or speech disturbances are uncommon and the patient or witness might take these symptoms as warning signs of some serious diseases leading to early arrival to the hospital. On the other hand, limb weakness, headache and sensory disturbances are not exclusive to stroke and they might take these symptoms as part of mild diseases or some psychiatric illnesses. Moreover, health seeking behavior among Nepalese people is poor [12, 13]. In a study related to cognitive and behavioral aspects of ischemic stroke, 59% of affected patients expected their symptoms to disappear spontaneously, and 25% of witnesses waited to see if the patient’s symptoms resolved spontaneously [14]. Studies have found that less than half of patients recognize they are having a stroke [14] or TIA [15], and some incorrectly attribute the symptoms to stress or fatigue, vision problems, migraine or heart attack [14, 15].

In our study, a visit to a local medical center was also associated with a late arrival, consistent with previous studies conducted in India and Taiwan [16, 17]. This is surprising since we expect medical professionals to readily detect the symptoms of a stroke and quickly refer the patient to the nearest tertiary hospital with thrombolysis service. Ours is one of the few hospitals in the city where a thrombolysis service is available, and lack of this information even among medical professionals can be a barrier to early referral. In our study, transportation by ambulance was not associated with prehospital delay. Studies have shown that the use of ambulances can shorten prehospital delay [18–20]. In our study, transfer by ambulance was observed only in 30.2% cases whereas this exceeds 50% in developed countries [21–23]. Emergency medical care system in Nepal is still in its infancy which is not readily available everywhere and is also considered expensive, and thus people prefer other modes of transportation for patient transfer. Heavy traffic, and the distance greater than 20 km was associated with delayed arrival to the emergency. Onset of a stroke at daytime was associated with an early arrival to the emergency. Our finding was in disagreement with other studies [21, 24]. This discrepancy can be explained by the fact that ambulance services and other modes of transportation are not always available and reliable at night in Nepal due to a limited number of ambulances, geographical condition and poor communication service of the country.

The onset of symptoms at home was not associated with prehospital delay in univariate analysis. However, it was independently associated with reduced prehospital delay in logistic regression model. While we do not know the exact reason, hesitancy, and concern for possible police inquiry may be seen as perceived barriers to rapid hospital transfer by the bystander. Diabetes mellitus was associated with delayed arrival to the emergency which is similar to observations made by Hong et al. and Song et al. [21, 23]. Symptoms of a stroke such as limb weakness, slurring speech, sensory disturbances, dizziness, and unconsciousness may be misinterpreted as autonomic disturbances following hypoglycemic episode. Such patients may involve in self-management and overlook or ignore new symptoms [25, 26].

Our study showed that a low income was associated with significant prehospital delay. Most of the medical care in Nepal, including emergency care, is paid out of pocket, often increasing the financial barrier to service utilization [27, 28]. Worse, even in the case of an emergency, the patient must pay first and then receive the service later. Health insurance coverage is minimal in Nepal, limited to a few cities and inaccessible to the poor. Nepal government launched a public health insurance plan in 2016/17, but it seems to be ineffective as it avails health services only up to $ 500 per head per year per family [28]. The limited amount of health cover discourages people to register for this plan. Poor financial status has several implications. It delays help seeking, transportation and affects decision making to undertake thrombolysis or not. Despite any contraindications, sixteen patients were deprived of thrombolysis even after reaching on time. Nepal is a low-income country, and recent data shows an annual per capita GDP of only $ 850. Alteplase is available as 50-mg vial in Nepal and costs 650 USD per 50-mg vial. Since the standard dose of alteplase for treatment of ischemic stroke is 0.9 mg/kg, a person weighing 55.5 kg or below requires one 50-mg vial and person weighing above 55.5 kg will require two 50-mg vial. Therefore, even after excluding all other laboratory, imaging, and therapeutic costs, the cost of thrombolysis alone using alteplase range from 650 USD to 1000 USD [29, 30]. This makes thrombolysis treatment exorbitant for poor patients. The increasing evidence for low-cost alternative tenecteplase offers hope for such patients [30–32]. In Nepal, tenecteplase is easily available as 30-mg vials which cost 450 USD. So the cost of thrombolysis using tenecteplase (Standard dose 0.25 mg/kg i.e. one 30-mg vial for 120 kg adult) will be around 450 USD, which is substantially affordable than using alteplase. Sometimes, if two ischemic stroke patients with the weight below 60 kg arrive at the same time, tenecteplase can even be shared between two patients to reduce the cost (225 USD). This has been practiced a few times at our center.

Many challenges exist for the patients, physicians, and healthcare systems, especially in low to middle income countries. With the rapidly changing face of healthcare in Nepal, some of these challenges can be tackled. Health promotion strategies to improve community awareness of early identification of symptoms of stroke, the importance of time window, rapid hospital transfer and encouraging the use of ambulance services may considerably reduce prehospital delay. Establishment of comprehensive stroke centers, developing central emergency medical services (EMS), and telestroke facility would go a long way in helping our cause. Telestroke can enable any trained physician located in tertiary hospital to conduct a rapid clinical assessment and can provide treatment recommendations for the local team of doctors [33]. In a meta-analysis conducted by Baratloo et al., telestroke significantly reduced onset-to-door and hospital-stay durations in stroke patients without increasing the risk of mortality or symptomatic intracranial hemorrhage [34]. However, at present, for establishing telestroke network, exorbitant set-up costs are required, which is prohibitive for the resource limited countries in which they are needed the most. But a recent study in India found that telestroke services based on smartphones are a cheaper alternative to video conferencing telestroke services and are more portable with fewer technical failures [35]. The government of Nepal can run a program to provide thrombolytic agents free of cost to poor and needy people. Justifiable use of tenecteplase can also help bring down the cost of thrombolysis.

The strength of this study is that we prospectively analyzed data for a relatively large sample from the largest tertiary hospital in Nepal. Our study is also one of the few reports on this issue in this part of the world and the first report of its kind from Nepal. However, the study has some limitations that need to be addressed. Firstly, this study was based on a single institute in the capital city. Thus, our result might not reflect the experience of patients with stroke in other cities and rural area. These limitations should be considered when interpreting the study results. Multicentered studies are required to assess the factors for the prehospital delay before we generalize these findings so that we can improve care for stroke patients.

## Conclusions

Prehospital delay is a major concern among stroke patients. Timely arrival to the hospital offers an opportunity for reperfusion therapy and good outcomes. Only 20% patients arrived in time frame for thrombolysis and 13.15% received thrombolysis. Many of the factors contributing to the delay in treatment after the onset of stroke can be overcome. We recommend community-based interventions to spread awareness about stroke and its related symptoms and the importance of timely hospital transfer in such patients. Concerned authorities should focus on the development of stroke centers, training specialists, and develop central emergency medical services. Use of low-cost tenecteplase as an alternative to alteplase should be encouraged to bring down the cost of reperfusion therapy. In resource limited settings, the establishment of telestroke facilities may be a viable option.

## Data Availability

The data sets used and analyzed in this study are available from the corresponding author on reasonable request.

## Abbreviations

CT: Computed tomography
DALY: Disability adjusted life years
ED: Emergency department
EMS: Emergency medical services
GDP: Gross domestic product
IVT: Intravenous thrombolysis
MRI: Magnetic resonance imaging
mRS: Modified rankin scale
NIHSS: National institute of health stroke scale
t-PA: Tissue plasminogen activator
USD: United stated dollars
